# Proinflammatory cytokine levels in fibromyalgia patients are independent of body mass index

**DOI:** 10.1186/1756-0500-3-156

**Published:** 2010-06-03

**Authors:** Maria E Hernandez, Enrique Becerril, Mayra Perez, Philippe Leff, Benito Anton, Sergio Estrada, Iris Estrada, Manuel Sarasa, Enrique Serrano, Lenin Pavon

**Affiliations:** 1 Department of Psychoimmunology, National Institute of Psychiatry "Ramon de la Fuente", Mexico; 2 Department of Immunology, National School of Biological Sciences, National Polytechnical Institute, Mexico; 3 Molecular Neurobiology and Addictive Neurochemistry Laboratory, National Institute of Psychiatry "Ramon de la Fuente", Mexico; 4 Araclon Biotech, SL, Clinica Montecanal (3a planta) C/Franz Schubert 2 E-50012 Zaragoza, Spain; 5 Department of Physical Activity Medicine, Universidad de Zaragoza, Spain

## Abstract

**Background:**

Fibromyalgia (FM) is characterized by chronic, widespread muscular pain and tenderness and is generally associated with other somatic and psychological symptoms. Further, circulatory levels of proinflammatory cytokines (IL-1β, TNF-α, and IL-6) may be altered in FM patients, possibly in association with their symptoms. Recently, rises in BMI have been suggested to contribute to increased circulating levels of proinflammatory cytokines in FM patients. Our aim was to measure the circulatory levels of proinflammatory cytokines to determine the influence of BMI on these levels in FM patients and healthy volunteers (HVs). In Spanish FM patients (n = 64) and HVs (n = 25), we measured BMI and serum concentrations of proinflammatory cytokines by capture ELISA.

**Findings:**

There were significant differences in BMI levels between FM patients (26.40 ± 4.46) and HVs (23.64 ± 3.45) and significant increase in IL-6 in FM patients (16.28 ± 8.13 vs 0.92 ± 0.32 pg/ml) (P < 0.001). IL-1β and TNF-α decreased in FM patients compared with HVs. By ANCOVA, there was no significant association between BMI and TNF-α (F = 0.098, p = 0.75) or IL-6 (F = 0.221, p = 0.63) levels in FM patients.

**Conclusions:**

Our analysis in FM patients of BMI as a covariate of proinflammatory cytokines levels showed that serum TNF-α and IL-6 levels are independent of BMI. Further studies are necessary to dissect these findings and their implication in future therapeutic approaches for FM patients.

## Introduction

Fibromyalgia (FM) is a common, chronic, widespread pain syndrome that primarily affects the joints and muscles and is generally associated with other somatic and psychological symptoms, including fatigue, poor sleep, cognitive difficulties, and stress [1]. FM patients are highly sensitive to painful and innocuous stimuli, including touch, heat, cold, chemicals, light, sound, and smell [2].

Although heightened pain sensitivity is a hallmark of FM, little is known about the factors (genetic and otherwise) that contribute to the development of this disease. Recently, some reports have noted alterations in proinflammatory cytokine levels in the serum and biopsies of FM patients [3,4], which might be associated with disease symptoms [5-7].

## Cytokines in fibromyalgia

Clinical studies have generated evidence that FM is associated with immune dysregulation of circulatory levels of proinflammatory cytokines, effecting the neural dysfunction of pain-related neurotransmitters [8]. Cytokines, depending on their concentration, induce symptoms, such as fatigue, fever, sleep, pain, and myalgia [9], all of which develop in FM patients.

Alterations in proinflammatory cytokine levels have been observed in the serum and biopsies of FM patients [3,4]. In addition, increased levels of IL-1Ra and IL-6 have been found in the supernatants of cells from FM patients in vitro stimulation and cellular proliferation studies [7]. Until recently, no infectious or degenerative event that was responsible for the variation in these levels had been identified in FM patients, although factors, such as obesity, can cause these alterations.

## BMI and inflammatory response

Obesity contributes to FM-related symptoms [10-14]. According to clinical guidelines that have been proposed by the National Institutes of Health, subjects are categorized as "normal" (BMI less than 25), "overweight" (BMI over 25 but less than 30), and "obese" (BMI greater than 30) [15].

Further, obesity alone is a risk factor for chronic pain disorders in general; for example, primary headaches are more common in obese individuals [16]. Additionally, recent evidence also suggests that obesity is characterized by a low-grade chronic inflammatory state, reflected by elevated levels in several serum inflammatory markers, such as interleukin-6 (IL-6) and C-reactive protein (CRP) [17].

The aim of this study was to measure circulatory levels of IL-1β, TNF-α, and IL-6 in FM patients and determine the influence of BMI as a covariate on the levels of these cytokines statistically.

## Materials and methods

The study design and protocol were reviewed and approved by the Ethics Committee of the Hospital Universitario Miguel Servet in Zaragoza, Spain, in accordance with the Declaration of Helsinki and the Nuremberg Code. All the patients in the study granted their informed consent.

### Fibromyalgia patients

Sixty-four female outpatients, aged 25 to 60 years (47.0 ± 8.48 years)--all of whom were members of the Aragonese Fibromyalgic and Chronic Fatigue Association (ASAFA) in Zaragoza, Spain--were recruited into the study.

At study entry, all recruited patients underwent a complete medical history, physical examination, and laboratory tests. The inclusion criterion was fibromyalgia that had been diagnosed 2 to 3 years ago, based on the current ACR diagnostic criteria [18]. Exclusion criteria were acute infectious diseases in the previous 3 weeks; past or present neurological, psychiatric, metabolic, autoimmune, allergy-related, dermatital or chronic inflammatory disease; medical conditions that required glucocorticoid treatment; past or current substance abuse or dependence; and pregnancy or current breastfeeding. Sixty-four potential participants met the inclusion criteria and were enrolled into the study.

### Healthy volunteers

Twenty-five healthy volunteers (HVs) were included in the study matching the age range (44.96 ± 10.01 years), gender, ethnicity (Spanish), and demographics (completion of at least 9 years of education and part of the middle socioeconomic class) of the recruited female FM subjects. HVs had no signs or symptoms of FM and were free of any medication for at least 3 weeks before the study began (based on blood and urine collection). HVs and FM patients submitted written informed consent before the studies began.

### BMI determinatión

Body mass index (BMI) was calculated for each participant (BMI = body weight [Kg]/height [m]^2^). Subjects were categorized as "normal" (BMI less than 25), "overweight" (BMI over 25 but less than 30), and "obese" (BMI greater than 30), per the clinical guidelines of the National Institutes of Health [15]. Body weight was measured using a standard Seca Beam Balance (Seca GmBH & Co Kg, Hamburg, Germany) as the reference instrument.

### Sample Collection

Blood was drawn between 8:00-9:00 A.M., and 20-mL blood samples were collected in separate sterile tubes that contained or lacked anti-clotting agents. Serum was obtained by centrifuging the blood samples at 272 × *g *for 10 min; 1-ml aliquots were stored at -70°C until use. These aliquots were used to measure the serum levels of cytokines. All samples were handled individually in a double-blind fashion.

### Cytokine quantification by capture ELISA

Quantitative cytokine assays were performed by standard capture enzyme-linked immunosorbent assay using Quantikine kits, for which the validated detection limits were 0.5-1000 pg/mL for TNF-α, 3.91-250 pg/mL for IL-1μ, and 0.7-300 pg/mL for IL-6. All kits, antibodies, and recombinant cytokines were purchased from R&D Systems (Minneapolis, MN). The assays were performed according to the manufacturer's instructions http://www.rndsystems.com, and absorbance was measured at λ = 492 nm on a spectrophotometer (Sunrise, Tecan). All samples were measured twice (in triplicate), from which the mean was calculated. The inter- and intra-assay variability was < 7% and < 5%, respectively.

### Statistical analysis

All values are expressed as mean ± standard deviation. Comparisons between healthy volunteers and patients were made by non parametric Mann-Whitney test for IL-6.

The student test (t) was used to analyze the differences in: a) BMI levels between healthy volunteers and patients; b) proinflammatory cytokine levels between BMI subsets (normal, overweight, and obese); and c) TNF-α levels between healthy volunteers and patients. Analysis of covariance (ANCOVA) was used to analyze the differences in proinflammatory cytokine levels and the covariate factor (BMI). The analysis was performed with Graph Pad (San Diego, CA, USA). In all tests, the null hypothesis was rejected at the 0.05 level.

## Results

### Clinical parameters

Clinical and laboratory assessments (i.e., complete blood count, blood chemistry, and complete urinalysis) of the fibromyalgia and control subjects fell within normal reference value ranges. No statistical differences were observed between the groups (data not shown).

### Cytokine measurements

The quantification of serum levels of proinflammatory cytokines in the FM and control groups by capture ELISA is shown in Table 1. IL-6 levels in FM patients were significantly higher than in HVs (Significance: p ≤0.001, Mann-Whitney test). TNF-α levels differed significantly between patients and HVs (t = 10.5, df = 87, p < 0.001). Although serum IL-1β levels were significantly lower in FM patients than in HVs, they were below the sensitivity levels, as indicated by the manufacturer (3.91 pg/mL); therefore, they were considered nondetectable (ND).

**Table 1:** Demographics; Body Mass Index; Cytokine levels in woman with fibromyalgia and Healthy volunteers

	Healthy Volunteers Female (n = 25)	FM Female (n = 64)	FM vs. Healthy Volunteers
Age (years)	44.96 ± 10.01	47.07 ± 8.48	N.S.
BMI (kg/m^2^)	23.64 ± 3.45	26.40 ± 4.46	*
TNF-α (pg/mL)	35.73 ± 0.72	20.42 ± 7.24	*
IL-1 (pg/mL)	17.04 ± 0.62	N.D.	N.A.
IL-6 (pg/mL)	0.92 ± 0.32	16.28 ± 8.13	*

Values are presented as mean (± SD) except where otherwise noted.

BMI = Body Mass Index; FM = Fibromyalgia patients; IL = Interleukin; N.D. = nondetectable; N.S. = not significant; N.A. = nonapplicable; TNF-α = Tumor Necrosis Factor - alpha.

t = p < 0.001

### BMI

The mean BMI values for the FM and HVs are shown in Table 1. Patients had significant BMI values with respect to HVs (t = 5.05, df = 87, p < 0.001). FM patients and HVs were classified as normal (FM = 24; HV = 17) and overweight (FM = 19; HV = 3). There were no significant differences in proinflammatory cytokine levels between BMI subsets (data not shown).

### Association between cytokine levels and BMI

Because BMI differed between HVs and FM patients, we performed ANCOVA to determine whether this covariable influenced cytokine levels. By ANCOVA, there was no significant association between BMI and TNF-α (F = 0.098. p = 0.75) or IL-6 (F = 0.221, p = 0.63) levels.

## Discussion

Currently, the diagnosis of fibromyalgia is reached after eliminating the possibility of rheumatic and psychiatric diseases, which represents a 2 or 3 year period before a specific diagnosis can be made. To attain a better understanding of this clinical condition and improve the quality of life for patients, we recommend that the variables that regulate FM be investigated, as well as the association between them. In this study, we measured proinflammatory cytokine levels and BMI.

Our study showed that FM patients had different circulatory levels of IL-6, TNF-α, and IL-1β compared with HVs. IL-6 increased significantly, which is consistent with other clinical studies [3]; further, other reports have noted an association of high levels of IL-6 (> 1 pg/mL) with painful events, surgical procedures and certain psychiatric disorders [19,20], as well as between fatigue and pain in animal models [21]; These findings suggests that IL-6 may regulates the clinical symptoms that develop in patients with FM.

TNF-α levels decreased in FM patients significantly in our study, in contrast to reports by Wang et al., Üçeyler et al., and Wallace et al., wherein TNF-α levels rose in untreated patients in one of the studies and were unchanged in the other reports [7,22,23]. This discrepancy might be due to variations in inclusion criteria for the patients, sample size, age, BMI, and detection methods [7,22,23].

In our study, IL-1β levels in FM patients were not within the range of detection, suggesting that they are significantly lower. Other reports, such as a study by Wallace et al., found that IL-1β levels in FM patients did not differ significantly from those in HVs, although levels of the soluble receptor for this cytokine (IL-1Ra) were significantly elevated [7]. These results suggest that IL-1Ra may induce a decrease in circulatory IL-1β to undetectable levels, as in our study.

The variations in cytokine levels in this study might reflect the activation of specific intracellular mechanisms. In healthy individuals, the levels of proinflammatory cytokines are controlled by diverse mechanisms, one of which involves the IL-6 receptor and gp130 protein. When IL-6 binds to its receptor, a mechanism is triggered blocking Janus Kinase signal and activator of transcription (JAK/STAT) mediated transcription of IL-1 and TNF-α [24], decreasing their levels in circulation [25,26]. This mechanism might explain the differential levels of proinflammatory cytokines that were observed.

Few studies have analyzed the association between BMI and cytokine levels in FM patients. One such study by Okifuji et al., reported a correlation between IL-6 levels and obesity, but 50% of their clinical samples comprised patients whose BMI ≥ 30 [11]. In contrast, we analyzed BMI as a covariate of variations in serum levels of IL-6 and TNF-α and found that ANCOVA test failed to reveal any significance, implying that the changes in cytokine levels were independent of BMI.

White adipose tissue regulates the production of TNF-α and IL-6, which induces low-grade inflammation [27,28]. Individual fat quantities can be inferred from waist circumference [15] but not BMI. The inclusion of waist circumference modifies the risk factor that is induced by the BMI with regard to the inflammatory and painful phenomena that are associated with obesity, such as fibromyalgia [13,16,29].

We conclude in this study, that variations in proinflammatory cytokines levels in FM patients are independents from BMI and the mechanisms underlying this response maybe a direct consequence of biochemical and functional alterations in FM patients. Further studies on fibromyalgia should consider BMI and waist circumference to develop a more accurate indicator of the amount of adipose tissue as a source of production of proinflammatory cytokines. The limitations of this study were the number of participants and the failure to measure waist circumference in the participants. Thus, to validate the diagnostic value of this clinical finding, a multicenter, longitudinal study must be performed in a large pool of homogeneous patients.

## List of Abbreviations

ACR: American College of Rheumatology; ANCOVA: analysis of covariance; ASAFA: Aragonese Fibromyalgyc and Chronic Fatigue Association; BMI: body mass index; CRP: C- reactive protein; FM: fibomyalgia patients; HVs: healthy volunteers; IL-1Ra: soluble receptor for inteleukin 1; IL: interleukin; ND: nondetectable; TNFα: interferon- alpha.

## Competing interests

MEH; EB; MP; PL; BA, SE; IE; ES and LP declare that they have no competing interests. MS is the current Chief Scientific Officer of Araclon Biotech.

## Authors' contributions

LP, MS, and ES designed the study and drafted the manuscript; ES supervised the enrollment of the subjects and participated in acquisition of the clinical data. All authors collected experimental data, participated in the analysis and interpretation of the data, reviewed the manuscript, and approved the final manuscript.
